# Recent Progress in Rice Broad-Spectrum Disease Resistance

**DOI:** 10.3390/ijms222111658

**Published:** 2021-10-28

**Authors:** Zhiquan Liu, Yujun Zhu, Huanbin Shi, Jiehua Qiu, Xinhua Ding, Yanjun Kou

**Affiliations:** 1 State Key Lab of Rice Biology, China National Rice Research Institute, Hangzhou 311400, China; lzq770799446@163.com (Z.L.); Zhuyujun@caas.cn (Y.Z.); shihuanbin@caas.cn (H.S.); qiujiehua@caas.cn (J.Q.); 2 State Key Laboratory of Crop Biology, Shandong Provincial Key Laboratory for Biology of Vegetable Diseases and Insect Pests, College of Plant Protection, Shandong Agricultural University, Taian 271018, China; xhding@sdau.edu.cn

**Keywords:** *Oryza sativa*, broad-spectrum resistance, rice blast, bacterial blight, breeding

## Abstract

Rice is one of the most important food crops in the world. However, stable rice production is constrained by various diseases, in particular rice blast, sheath blight, bacterial blight, and virus diseases. Breeding and cultivation of resistant rice varieties is the most effective method to control the infection of pathogens. Exploitation and utilization of the genetic determinants of broad-spectrum resistance represent a desired way to improve the resistance of susceptible rice varieties. Recently, researchers have focused on the identification of rice broad-spectrum disease resistance genes, which include *R* genes, defense-regulator genes, and quantitative trait loci (QTL) against two or more pathogen species or many isolates of the same pathogen species. The cloning of broad-spectrum disease resistance genes and understanding their underlying mechanisms not only provide new genetic resources for breeding broad-spectrum rice varieties, but also promote the development of new disease resistance breeding strategies, such as editing susceptibility and executor *R* genes. In this review, the most recent advances in the identification of broad-spectrum disease resistance genes in rice and their application in crop improvement through biotechnology approaches during the past 10 years are summarized.

## 1. Introduction

Rice (*Oryza sativa* L.) is the most important food crop, which is consumed by approximately 50% of the world’s population, with its consumption growing dramatically in many parts of the world. Stable rice production is constrained by various biotic stresses, including fungal blast caused by *Magnaporthe oryzae*, sheath blight caused by *Rhizoctonia solani*, false smut caused by *Ustilaginoidea virens*, bakanae disease due to *Fusarium fujikuroi*, bacterial blight caused by *Xanthomonas oryzae pv. oryzae* (*Xoo*), bacterial leaf streak caused by *Xanthomonas oryzae pv. oryzicola* (*Xoc*), and virus diseases. The yield loss of rice caused by various diseases averages upward of 30%. Therefore, it is critical to adopt effective means to control these diseases to ensure global food security. In addition to encouraging farmers to exercise good farming practices, application of pesticides remains one of the main methods of disease control, but the increase in costs and their harmful effects on the environment and operators cannot be discounted. These make the farmers largely dependent on the cultivation of new resistant varieties, which is considered to be the most effective method so far.

Broad-spectrum resistance, which refers to resistance against two or more types of pathogen species or the majority of races/isolates of the same pathogen species, is one of the ultimate goals of breeders for rice improvement [1]. Exploitation of the genetic determinants of broad-spectrum resistance will improve the resistance of the susceptible varieties. On this account, breeders and biotechnologists are trying to obtain the source of broad-spectrum resistance to understand and utilize the genetics underlying this process. With the development of rice molecular biology, functional genomics, and gene editing technology, great progress has been made in the broad-spectrum disease resistance genes in rice. It is worth mentioning that many extremely important broad-spectrum disease resistance genes and their mechanisms and applications were reported 10 years ago. These works have been well summarized in several reviews and will not be highlighted here [1,2]. This review focuses on the progress in the identification of broad-spectrum disease resistance genes in rice and their application in crop improvement during the past 10 years.

## 2. Identified Rice Broad-Spectrum Disease Resistance Genes in Past 10 Years

### 2.1. R Genes Confer Broad-Spectrum Disease Resistance in Rice

The ability of plants to defend themselves against microbes is specified by disease *resistance* (*R*) genes-mediated resistance and basal resistance. Upon recognition of an invading pathogen, R proteins, mostly from the nucleotide-binding leucine-rich repeat receptor (NLR) family, detect the secreted pathogen effectors to activate a multitude of responses that ultimately lead to resistance. These responses include Ca^2+^ influx, ROS (reactive oxygen species) accumulation, mitogen-activated protein (MAP) kinase activation, defense hormone signaling activation, and upregulation of defense-related genes [3]. In recent years, important progress has been achieved in cloning and mechanical analysis of *R* genes. These identified *R* genes provide not only new genetic resources for breeding broad-spectrum rice varieties, but also new strategies to improve resistance.

**Figure 1:**
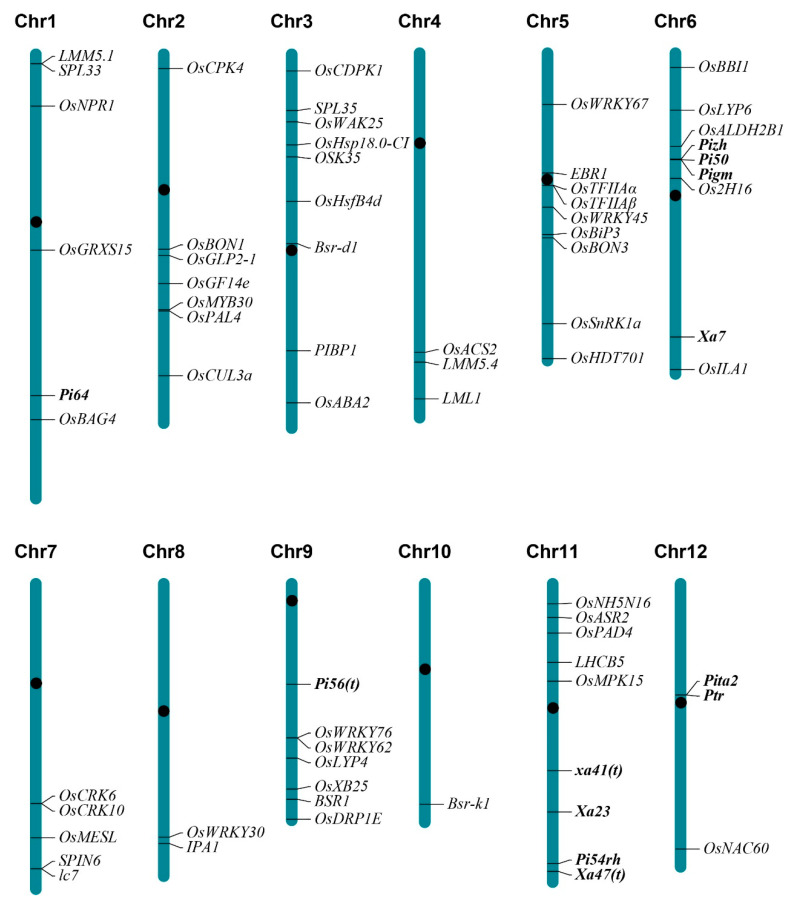
*R* and defense regulator genes with broad-spectrum disease resistance identified in past 10 years. The *R* genes are represented in bold black font. The black dots on each chromosome represent centromeres.

Rice blast, caused by *M. oryzae*, is the most devastating disease of rice and reduces yield by 10–35%. To date, approximately 100 *R* genes/alleles against *M. oryzae* have been identified, of which more than 26 *R* genes/alleles were cloned [2]. Among these genes, *Pi50*, *Pi54rh*, *Pi56*(*t*), *Pi64*, *Pigm*, *Pizh*, *Ptr*, and *Pita2* were cloned as broad-spectrum resistance *R* genes in the past 10 years [4,5,6,7,8,9,10] (Figure 1, Table 1). *Pi50*, *Pigm*, and *Pizh* are allelic to *Pi2/Pi9*, which are well-known broad-spectrum resistance *NBS-LRR* (*nucleotide binding site-leucine rich repeat*) genes on the chromosome 6, with different resistance spectra against *M. oryzae*. One of these alleles *Pigm* has been confirmed with stable resistance to panicle blast [7]. *Pi54rh*, an ortholog of *Pi54*, encodes an NBS-LRR protein with a unique Zinc finger domain. Both *Pi56*(*t*) and *Pi64* also belong to the NBS-LRR family of disease resistance genes. Notably, the constitutively expressed *Pi64* conferred resistance to both leaf and neck blast. Unlike most blast *R* genes, *Ptr*, which is required for broad-spectrum blast resistance mediated by *R* genes *Pita* and *Pita2*, encodes a four Armadillo (ARM) repeat protein. Furthermore, more alleles or natural variation of broad-spectrum blast resistance *R* genes have been investigated, including geographically distinct and domain-specific sequence variations of *Pib*, novel alleles of *Pik* locus *Pi1*, *Pike,* and *Pikg*, *Pi54* alleles, novel *Pi21* haplotypes, and novel alleles of *Pi2/9* locus [11,12,13,14,15,16,17,18,19,20,21,22]. In addition, four broad-spectrum resistance *R* genes, *Pi-hk1*, *Pi57*(*t*), *Pi65*(*t*), and *Pi69*(*t*), were finely mapped in the past 10 years [23,24,25,26]. 

In addition to rice blast, bacterial blight, caused by *Xoo*, is also a globally devastating rice disease. In rice, at least 46 genes that confer dominant or recessive host resistance to *Xoo* have been identified, of which more than 16 *R* genes were cloned [27]. Among them, *Xa7*, *Xa23*, *Xa41*(*t*), and *Xa47*(*t*) were cloned as broad-spectrum resistance *R* genes in the past 10 years [27] (Figure 1, Table 1). *Xa7*, which encodes a 113 aa unknown protein, is a dominant *R* gene that provides broad-spectrum and extremely durable resistance to *Xoo*. The transcription of *Xa7* is specifically activated by the *Xoo* isolates with transcription activator-like effector (TALE) AvrXa7 or PthXo3 to act as an executor [27]. Another executor *R* gene, *Xa23*, which is induced by TALE AvrXa23, confers extremely broad-spectrum resistance to *Xoo* [28]. *xa41*(*t*), an allele of sugar transporter *OsSWEET14*, confers resistance to half of the tested *Xoo* isolates [29]. Similar to *Xa23*, *Xa47*(*t*) is from the wild rice *Oryza rufipogon. Xa47*(*t*), encoding a NLR protein, is highly resistant to all tested ten *Xoo* isolates [30]. In addition to these cloned *R* genes, a broad-spectrum bacterial blight resistance gene *Xa33* from *Oryza nivara* has been finely mapped [31].

Unlike rice blast and bacterial blight, no *R* gene for serious diseases sheath blight and rice false smut has been identified [32,33]. Moreover, only one rice stripe virus (RVS) resistance gene, *STV11*, has been cloned [34]. The molecular mechanisms underlying rice–virus interaction remain poorly understood. Therefore, so far, there is no broad-spectrum resistance *R* gene for these diseases. 

For broad-spectrum resistance *R* genes, it is worth noting that the *R* genes are tagged as broad-spectrum resistance genes because they can resist multiple isolates of one pathogen rather than two or more types of pathogen species. However, in the case of many isolates tested, it is unlikely for any *R* gene to be resistant to only one isolate. At present, there is no standard in terms of how many isolates or what proportion of isolates an *R* gene confers resistance to for it to be claimed as a broad-spectrum resistance *R* gene. Furthermore, although so many broad-spectrum resistance genes have been identified, the mechanism of these genes mediating broad-spectrum resistance to rice disease is not yet clear. In the broad-spectrum resistance *R* gene *Pi9* case, the corresponding *Avirulence* gene *AvrPi9* exists widely in various *M. oryzae* isolates [35]. To determine whether other blast *R* genes are similar, the cloning of their corresponding *Avirulence* genes and analysis of their distribution in *M. oryzae* isolates will give some hints. Similar to *R* genes against blast, the resistance spectrum of *R* genes against *Xoo* may also be determined by the distribution of corresponding *Avirulence* genes, *TALE* in most of cases, in *Xoo* isolates. Actually, considering current knowledge, it is difficult to predict the mechanism underlying R protein-mediated broad-spectrum resistance in addition to the wide distribution of corresponding *Avirulence* genes. The research progress of the interactions between R protein and effectors from pathogens will increase our understanding of R protein-mediated broad-spectrum resistance.

### 2.2. Defense Regulator Genes Contribute to Broad-Spectrum Disease Resistance

Differing from *R* genes, defense *regulator* genes often confer partial resistance to a broad spectrum of pathogen isolates or various pathogens. In the past 10 years, there were at least 56 broad-spectrum resistant defense regulator genes identified which positively or negatively regulate the resistance to rice diseases (Figure 1, listed in Table 2). The proteins encoded by these genes are transcriptional factors, kinases, peroxidases, E3 ubiquitin ligases, ferredoxin-dependent glutamate synthases, glutaredoxins, etc. In this review, we classify these broad-spectrum resistant defense regulator genes according to the types of pathogens they resist.

In the past 10 years, several broad-spectrum resistant defense regulator genes against *M. oryzae* were identified. Through a genome-wide association study (GWAS), a natural allele of a C2H2-type transcription factor *bsr-d1* was identified in rice that confers non-race-specific resistance to blast. This allele causes a lower gene expression level, and then downregulates expression of three peroxidase-encoding genes, *Os05g04470*, *Os10g39170*, and *Perox3*, resulting in accumulation of H_2_O_2_ and enhanced broad-spectrum resistance to *M. oryzae* [36,37]. In addition, an MYB transcription factor (OsMYB30) is also involved in *bsr-d1*-mediated broad-spectrum blast resistance by activating the lignin biosynthesis genes Os4CL3 and *Os4CL5* to strengthen cell walls [38]. The other three transcription factors, OsNAC60, OsWRKY45, and RRM (RNA recognition motif) protein PIBP1 (PigmR-interacting and blast resistance protein 1), also contribute to broad-spectrum blast resistance in rice. *OsNAC60*, which is a target of Osa-miR164a, negatively regulates rice immunity against the blast fungus *M. oryzae* by decreasing programmed cell death, ion leakage, ROS accumulation, callose deposition, and defense-related gene expression [39]. OsWRKY45 mediates the blast resistance of CC-NB-LRR protein Pb1 [40]. PIBP1 specifically interacts with PigmR and other similar NLRs, and it functions as an unconventional transcription factor to activate the expression of OsWAK14 and *OsPAL1* to trigger blast resistance [41]. In addition to transcription factors, the RING protein OsBBI1 with E3 ligase activity and light-harvesting complex II protein LHCB5 are also involved in broad-spectrum blast resistance. OsBBI1 confers broad-spectrum resistance against *M. oryzae* by increasing H_2_O_2_ accumulation in cells and modifying the cell-wall defense [42]. Phosphorylation of LHCB5 enhances resistance to *M. oryzae* through the accumulation of ROS in the chloroplast [43]. 

Several broad-spectrum resistant defense-regulator genes against *Xoo* were also identified in rice in the past 10 years. In rice, several genes involved in receptor-mediated broad-spectrum resistance and systemic acquired resistance (SAR) likely contribute to broad-spectrum resistance to *Xoo*. The XA21-binding protein XB25, a plant-specific ankyrin repeat (PANK) protein, contributes to the accumulation of receptor XA21 and maintenance of XA21-mediated broad-spectrum resistance to *Xoo* [44]. The endoplasmic reticulum (ER) chaperone, luminal-binding protein 3 (BiP3) negatively regulates resistance mediated by rice XA3, a receptor that provides broad-spectrum resistance to *Xoo* [45]. Overexpression of *OsNPR1* *(non-expressor of pathogenesis-related genes 1*), a master gene for SAR in rice, greatly enhances resistance to *Xoo* [46]. Moreover, the cysteine-rich-receptor-like kinases (OsCRK6 and OsCRK10) are required for *OsNPR1*-mediated immunity [47]. In addition to CRK6 and CRK10, some kinases have been identified as conferring broad-spectrum resistance to *Xoo*. For instance, overexpression of a constitutively activated form of calcium-dependent protein kinases OsCDPK1 confers *Xoo* resistance by affecting *OsPR10a* expression in rice [48]. OsILA1, a Raf-like MAPKKK, functions as a negative regulator and acts upstream of the OsMAPKK4–OsMAPK6 cascade against *Xoo* [49]. Unlike the type of genes mentioned above, *lc7*, encoding a mutant ferredoxin-dependent glutamate synthase 1 (Fd-GOGAT1), promotes ROS accumulation in the leaves and has high broad-spectrum resistance against seven *Xoo* strains [50].

Defense regulator genes are different from pathogen-specific *R* genes, which can confer resistance to multiple pathogens. For instance, Lysin motif-containing protein genes *LYP4* and *LYP6*, transcriptional regulator genes *OsWRKY67* and *IPA1*, the host basal transcription factor IIA subunit genes *OsTFIIA*α and *OsTFIIA*β, germin-like protein gene *OsGLP2*-1, sucrose nonfermenting 1-related protein kinase 1 genes *OsSnRK1a* and *OsSnRK1b*/*OSK35*, calcium-dependent protein kinase gene *OsCPK4*, and receptor-like cytoplasmic kinase gene *broad-spectrum resistance 1 (BSR1)* play a positive role in basal resistance against *M. oryzae* and *Xoo* [51,52,53,54,55,56,57,58,59,60]. In contrast, mutations in E3 ubiquitin ligase gene *EBR1* (enhanced blight and blast resistance 1), RhoGAP *SPIN6*, rice wall-associated kinase gene *OsWAK25*, Cullin 3-based RING E3 ligase gene *OsCUL3a*, dynamin-related protein gene *OsDRP1E*, eEF1A-like protein gene *SPL33*, eukaryotic translation elongation factor 1A-like genes *LMM5*.1 and *LMM5*.4, eukaryotic release factor 1 gene *LMM1*, *abscisic acid 2 (OsABA2)*, and CUE domain-containing protein gene *SPL35* result in lesion mimic leaves and enhanced broad-spectrum resistance to *M. oryzae* and *Xoo* [61,62,63,64,65,66,67,68,69,70]. Differing from these lesion mimic genes, histone H4 deacetylase gene *HDT701* and mitogen-activated protein kinase *OsMPK15*, whose mutant or overexpressing lines do not show lesion mimic leaves, negatively regulate the resistance against *M. oryzae* and *Xoo* [71,72]. Similarly, loss of function of the *Bsr-k1* gene, encoding a tetratricopeptide repeat (TPR)-containing protein, leads to accumulation of *OsPAL1–7* mRNAs, which confer broad-spectrum resistance against *M. oryzae* and *Xoo* with no major penalty on key agronomic traits [73]. 

In addition, several genes have been reported as conferring broad-spectrum resistance against multiple pathogens other than both *M. oryzae* and *Xoo*. Aldehyde dehydrogenase OsALDH2B1 has a moonlight function as a transcriptional regulator to regulate a diverse range of biological processes involving G protein, brassinolide, jasmonic acid, and salicylic acid signaling pathways. Loss of function of *OsALDH2B1* greatly enhanced resistance to *M. oryzae*, *Xoo*, and *Xoc* [74]. Similarly, rice phenylalanine ammonia-lyase gene *OsPAL4* is associated with resistance to *M. oryzae*, *Xoo*, and *Xoc* [75]. Heat-shock factor OsHsfB4d binds the promoter and regulates the expression of a small heat-shock protein gene *OsHsp18.0-CI* to be resistant against *Xoo* and *Xoc* [76,77]. Moreover, suppression of *phytoalesin-deficient 4 OsPAD4* results in increased susceptibility to the *Xoo* and *Xoc* [78]. Rice glutaredoxin gene *OsGRXS15* and a novel *NPR1* homolog gene *OsNH5N16* contribute to broad-spectrum resistance to *Xoo* and *F. fujikuroi* by regulating the expression of *PR* genes related to SAR [79,80]. *Abscisic acid*, *stress, and ripening 2 (ASR2)* contributes to broad-spectrum resistance against *Xoo* and *R. solani* by regulating the expression of a defense regulator gene *Os2H16* [81,82]. In contrast, 14-3-3 protein (GF14e) negatively affects cell death and disease resistance to *Xoo* and *R. solani* [83]. WRKY transcription factor *OsWRKY30* and 1-aminocyclopropane-1-carboxylic acid synthase gene *ACS2* positively regulate the resistance against *M. oryzae* and *R. solani* [84,85]. Moreover, methyl esterase-like gene *OsMESL* and copine genes *OsBON1* and *OsBON3* are critical suppressors of immunity to *M. oryzae*, *Xoo*, and *R. solani* [86,87]. 

These excellent studies on broad-spectrum resistant defense regulator genes show multiple characteristics. Firstly, with the increasing attention to broad-spectrum resistance, the reports of broad-spectrum resistance related genes have increased sharply in the past 10 years [1]. For breeders, whether these genes also show broad-spectrum disease resistance in the natural field environment is still the focus of attention. Secondly, the connections between these broad-spectrum resistant defense regulator genes and their relationships with *R* genes remain largely unclear due to limited experimental evidence. It is possible that these broad-spectrum resistance defense regulator genes function in the convergence point of the crosstalk between the pathways of basal and R protein-mediated resistances or between the pathways initiated by different R proteins [1]. Thirdly, only a few broad-spectrum resistant defense regulator genes mediate resistance with little or no yield penalties. The tradeoff between broad-spectrum resistant defense regulator genes and rice yield is one of the important limiting factors, as summarized in Chen’s review [2]. Last but not least, there are rare examples of using these disease resistance-related genes to obtain broad-spectrum disease-resistant varieties in breeding programs. Although it was very difficult to effectively use these broad-spectrum resistant defense regulator genes in molecular breeding of rice until now, identification of natural variations/alleles of these genes from rice varieties with excellent agronomic traits, artificial mutation, and genome-editing technology would provide important methods for broad-spectrum disease resistance.

### 2.3. Identification of Broad-Spectrum Disease Resistance Loci by QTL Mapping and GWAS Analysis

Broad-spectrum resistance is a polygenic trait, whereby a combinatorial effect of major and minor genes mediates this trait [88]. With the advances of next-generation DNA sequencing and high-density molecular marker platforms, various quantitative trait loci (QTL) against rice blast, sheath blight, and/or bacterial leaf blight have been mapped to locate the source of these traits in the past 10 years. Using Heikezijing, a *japonica* rice landrace with broad resistance against rice blast and Suyunuo recombinant inbred lines, 13 QTLs were identified to be effective against only one *M. oryzae* isolate, while the other seven QTLs may be non-isolate-specific because each QTL confers resistance to more than one isolate [89]. By evaluating the disease reactions of 60 US weedy rice accessions with 14 *M. oryzae* isolates, 28 resistant QTLs were identified, of which three loci contribute to non-isolate-specific resistance [90]. With a combination of genome-wide association studies (GWAS) and interval mapping analyses, 51 QTLs against *Xoo* and *Xoc* were identified in multiparent advanced generation intercross populations, including 11 broad-spectrum resistance, three pathovar-specific, and 37 isolate-specific QTLs [88]. A GWAS analysis of 236 diverse rice accessions revealed 12 QTLs, of which two QTLs showed broad-spectrum resistance to *Xoc* [91]. Recently, 147 SNP associated with *Xoo* resistance were identified in 222 predominantly Thai rice accessions; however, the significantly associated SNP only occurred across a maximum of five *Xoo* isolates [92]. In rice, most resistance QTLs are conditioned to different populations and different QTL mapping analyses, which makes it difficult to handpick suitable QTL candidates for breeding programs with multiple resistances. To integrate QTL from different studies, a meta-analysis of QTLs represents a good approach. Using meta-analysis, 48 meta-QTLs were obtained from 27 studies, of which MQTL8.1 and MGTL2.5 were associated with resistance to rice blast, sheath blight, and bacterial leaf blight [93]. Resistant QTLs confer a partial but frequently referred resistance to broad-spectrum pathogen isolates or diverse pathogens, which are considered as effective resources for breeding to achieve broad-spectrum resistance [1]. However, the results from all these studies clearly showed that most QTLs confer isolate- and/or pathogen-specific resistance; in other words, not all resistance QTLs are broad-spectrum. Moreover, there are few broad-spectrum resistant QTLs available for crop improvement programs, which results in the breeder needing a longer time and higher cost to pyramid QTLs to obtain broad-spectrum disease-resistant varieties.

## 3. Strategies for Broad-Spectrum Disease Resistance Rice Breeding

### 3.1. Gene Pyramiding Breeding Is an Effective Way to Obtain Broad-Spectrum Disease Resistance Rice Varieties

Developing and using resistant varieties could effectively and economically control diseases. One of the effective ways to develop broad-spectrum resistance varieties is marker-assisted gene pyramiding. Numerous cloned *R* genes provide a wealth of information and resources for pyramiding breeding, which promoted the generation of pyramiding *R* gene lines with broader and enhanced resistance to bacterial blight and rice blast in the past 10 years. Introducing *Piz*/*Pi2*/*Pi9*, *Pid3*, or *Pi54* or pyramiding the *R* genes *Pi37* + *Pid3*, *Pi5* + *Pi54*, *Pi54* + *Pid3*, *Pigm* + *Pi37*, *Pi9* + *Pi54*, *Pigm* + *Pi1*, *Pigm* + *Pi33*, *Pigm* + *Pi54*, *Pi2* + *Pi46* + *Pita*, *Pi2* + *Pi46* + *Pigm*, and *Pib* + *Pi25* + *Pi54* leads to broad-spectrum blast resistance [94,95,96,97,98,99,100,101]. Pyramiding the *R* genes *Xa4* + *xa5* + *Xa21*, *xa5* + *xa13* + *Xa21*, *xa5* + *Xa21*, *Xa21* + *Xa33*, and *Xa23* with other genes, as well as *Xa4* + *xa5* + *Xa7* + *xa13* + *Xa21*, provides a higher and broader resistance to *Xoo* than individual resistance genes [102,103,104,105,106,107,108]. Moreover, pyramiding the genes *Pi2* + *Xa7* and *xa5* + *xa13* + *Pi54* + *qSBR7-1* + *qSBR11-1* + *qSBR11-2* confers broad-spectrum resistance to both *M. oryzae* and *Xoo* [109,110].

All aforementioned studies give excellent examples of pyramiding *R* genes to obtain broad-spectrum resistance rice. However, it is still very difficult to obtain broad-spectrum disease resistant varieties by polymerization breeding for several reasons [111,112]. The first is that only a few *R* genes have been successfully used for molecular breeding processes. The existence of *R* genes containing resistant germplasms with excellent comprehensive traits is an important perquisite for breeding application. Secondly, the utilization of *R* genes in main modern rice varieties and the effectiveness of *R* genes in different rice-cultivating regions are still not very clear. Thirdly, the resistance effect of pyramiding different *R* genes may not be a simple accumulation of resistance spectrum and improvement in the resistance level; meanwhile, with the increase in the number of pyramided *R* genes via traditional genetics approaches, the workload of breeding, time consumption, and linkage drag with unacceptable traits increase. Therefore, an accurate understanding of *R* gene utilization and the establishment of high-throughput molecular breeding methods to create *R* genes harboring resistant germplasms without linkage drag are important steps to overcome these difficult points, so as to improve broad-spectrum resistance breeding in the future.

### 3.2. Engineering Broad-Spectrum Disease Resistance Rice by Editing Susceptibility and Executor R Genes Is a New Method with Broad Application Prospects

During the plant–pathogen interaction, phytopathogens evolve to exploit the *susceptibility (S)* genes of plant to facilitate their infection. These *S* genes are associated with host recognition, penetration, pathogen proliferation and spread, or negative regulation of immune signals [113]. Disrupting these *S* genes can lead to enhanced resistance or reduced compatibility and, consequently, expand resources for broad-spectrum disease resistance. To date, many *S* genes have been identified in rice, such as *Pi21*, *Xa5,* *Xa13/OsSWEET11*, *Xa25/OsSWEET13*, and *Xa41*/*OsSWEET14* [29,114,115,116,117,118,119,120,121]. Recent advances in genome editing technologies, such as the CRISPR (clustered regularly interspaced short palindromic repeats)/Cas9 (CRISPR-associated protein 9)-mediated gene editing system, have greatly accelerated the generation of new resistant rice through genetic manipulation of *S* genes [113,122].

**Figure 2:**
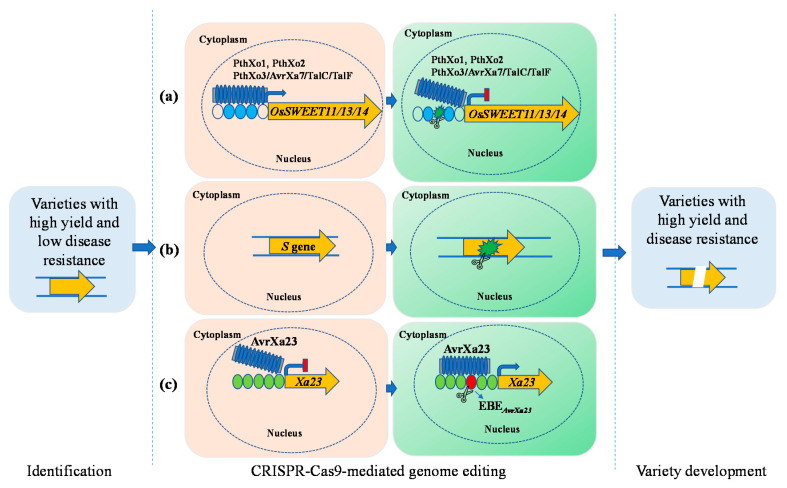
Engineering broad-spectrum disease-resistant rice by editing susceptibility and executor *R* genes. (**a**) Editing the *EBEs* (effector-binding elements) of *S* genes *OsSWEET11*, *OsSWEET13*, and *OsSWEET14*; (**b**) disruption of *S* genes; (**c**) editing the *EBEs* of executor *R* gene by CRISPR/Cas9-mediated precise homology directed repair.

The most reported examples of editing *S* genes in rice can be found in research related to *Xoo* resistance. During the infection process of *Xoo*, abundant transcription activator-like effectors (TALEs), which are the major virulence factors and compatibility determinants, are secreted into rice cells. Most TALEs bind to the *cis*-element effector-binding elements (*EBEs*) in the promoter of *S* gene and reprogram their transcription to promote disease. For instance, the TALEs PthXo1, PthXo2, and PthXo3/*AvrXa7*/TalC/TalF bind the *EBEs* in the promoters of rice *OsSWEET11*, *OsSWEET13*, and *OsSWEET14* genes, respectively (Figure 2a) [122]. Editing the *EBEs* of *S* genes *OsSWEET11*, *OsSWEET13*, and *OsSWEET14* in rice varieties japonica Kitaake and indica IR64 and Ciherang-sub1 resulted in loss of induction of these *S* genes by *Xoo* and broad-spectrum resistance against *Xoo* [123,124,125,126]. Similar strategies were used in the modification of *S* genes, *Pi21*, *Bsr-d1*, and *Xa5* to obtain broad-spectrum resistance rice against *Xoo* and *M. oryzae* [127]. In addition, it was well summarized that editing the open reading frame of *susceptibility defense regulators* could obtain broad-spectrum resistance rice plants in the Wang’s review (Figure 2b) [128]. 

Contrary to the interaction between TALEs and rice *S* genes, the usually suppressed executor *R* genes, such as *Xa10* and *Xa23*, are transcriptionally activated by TALEs to restrict the growth of *Xoo* [28]. Using an in-depth understanding of the mechanism underlying the interaction between TALEs and executor *R* genes, a new strategy for engineering broad-spectrum bacterial blight resistance through CRISPR/Cas9-mediated precise homology directed repair was proposed. Using this strategy, the EBE_*AvrXa23*_, which is bound by TALE *AvrXa23* to activate the expression of *Xa23*, was inserted into the promoter region of the susceptible *xa23* allele in the susceptible rice cultivar, resulting in a resistant variety (Figure 2c) [129]. This is a significant expansion to the application of executor *R* genes and a new genome editing strategy in improving rice disease resistance. 

### 3.3. Transgenic Rice Expressing Genes from Other Species Shows Broad-Spectrum Disease Resistance

Along with pyramiding resistance genes and editing susceptibility genes in rice, development of transgenic rice plants by expressing defense genes from other species is an appropriate approach to control pathogens, especial in the absence of a resistant germplasm. For instance, the *Arabidopsis* NPR1 protein (non-expressor of PR1) is a key regulator in the signal transduction pathway leading to the activation of SAR, which is a broad-spectrum resistance response upon exposure to a pathogen [130]. Constitutive expression of the *AtNPR1* gene in rice leads to high resistance but growth and agronomic trait defects. To overcome this problem, different strategies were developed by two groups [130,131]. Eventually, broad-spectrum resistant rice plants without a fitness cost were obtained by expression of *AtNPR1* driven by green tissue-specific promoter or pathogen-responsive upstream open reading frames of key immune regulators TBF1 [130,131]. Similarly, transgenic rice lines expressing the auto-activated *NLR* genes *RPS2* and *RPM1* (D505V) from *Arabidopsis* conferred broad-spectrum resistance to pathogens *M. oryzae* and *Xoo* via early and strong induction of ROS, callose deposition, and expression of defense-related genes. These *RPS2* and *RPM1* cases revealed that auto-activated NLRs are a promising resource for breeding crops with broad-spectrum resistance, and they provide new insights for engineering disease resistance [132]. In addition to genes from *Arabidopsis*, transgenic rice plants expressing resistant *Lr34* allele from wheat showed increased resistance against multiple isolates of the hemibiotrophic pathogen *M. oryzae* by delaying invasive hyphal growth [133]. In another example, transgenic rice lines expressing the isoflavone synthase (GmIFS1) gene from soybean contributed to the synthesis of isoflavone (genistein) to promote *M. oryzae* resistance, indicating that the synthesis of heterologous secondary metabolites, such as isoflavone, is a good way to develop blast resistance in rice [134]. As such, we believe that engineering resistant rice through ectopic transcription of defense genes cloud be a broadly applicable new strategy, which may lead to reduced use of pesticides and lightening the selection pressure of resistance pathogens.

## 4. Conclusions

In the past 10 years, several broad-spectrum *R* genes, defense regulators, and QTLs were identified in rice with resistance against two or more types of pathogen species or many isolates of the same pathogen species. This emerging knowledge of broad-spectrum resistance genes formulates efficient ways to best use these genetic resources for crop improvement via biotechnological approaches. However, there are still many gaps in our knowledge of the mechanisms underlying broad-spectrum resistance. To reveal these mechanisms, more research about the interactions between the host R protein and pathogen effectors, as well as the cooperation among these broad-spectrum resistance genes, is required in future. Furthermore, there is still a long way to using these genes to create broad-spectrum disease-resistant varieties. An accurate understanding of *R* gene utilization, the establishment of high-throughput molecular breeding methods to create *R* genes harboring resistant germplasms without linkage drag, and an investigation of new strategies for using defense regulator genes without a yield penalty will be helpful for improving broad-spectrum resistance breeding in the future.

## Figures and Tables

**Table 1:** *R* genes with broad-spectrum disease resistance in rice reported in past 10 years.

Gene Name	Protein Type	Isolates or Pathogens ^1^	Chromosome	Reference
*Pi64*	NBS-LRR	9 *Mo* isolates	1	[6]
*Pizh*	NBS-LRR	31 *Mo* isolates	6	[8]
*Pigm*	NBS-LRR	30 *Mo* isolates	6	[7]
*Pi50*	NBS-LRR	20 *Mo* isolates	6	[22]
*Pi56*(*t*)	NBS-LRR	19 *Mo* isolates	9	[5]
*Pi54rh*	NBS-LRR	7 *Mo* isolates	11	[4]
*Ptr*	ARM repeat protein	331 *Mo* isolates	12	[9]
*Pita2*	ARM repeat protein	64 *Mo* isolates	12	[10]
*Xa7*	Executor R protein	8 *Xoo* isolates	6	[27]
*Xa23*	Executor R protein	39 *Xoo* isolates	11	[28]
*xa41*(*t*)	Sugar transporter (SWEET)	18 *Xoo* isolates	11	[29]
*Xa47*(*t*)	NBS-LRR	10 *Xoo* isolates	11	[30]

^1^ The pathogens and number of isolates to which resistance is conferred by the gene in the first column. *Magnaporthe oryzae*, *Mo*; *Xanthomonas oryzae pv. oryzae*, *Xoo*.

**Table 2:** Defense regulator genes showing broad-spectrum disease resistance.

Gene Name	Protein Type	Isolates or Pathogens ^1^	Chr ^2^	Reference
*Bsr-d1*	C2H2-type transcription factor	9 *Mo* isolates	3	[36,37]
*OsMYB30*	MYB transcription factor	5 *Mo* isolates	2	[38]
*OsNAC60*	NAC transcription factor	20 *Mo* isolates	12	[39]
*OsWRKY45*	WRKY transcription factor	1 *Mo* isolate	5	[40]
*PIBP1*	RRM (RNA recognition motif) protein	3 *Mo* isolates	3	[41]
*OsBBI1*	RING E3 ubiquitin ligase	7 *Mo* isolates	6	[42]
*LHCB5*	Light-harvesting complex II protein	21 *Mo* isolates	11	[43]
*OsXB25*	Plant-specific ankyrin-repeat (PANK) protein	1 *Xoo* isolate	9	[44]
*OsBiP3*	Endoplasmic reticulum (ER) chaperone, luminal-binding protein 3	2 *Xoo* isolates	5	[45]
*OsNPR1*	BTB/POZ-ankyrin repeat protein	1 *Mo* isolate, 2 *Xoo* isolates	1	[46]
*OsCRK6*	Cysteine-rich-receptor-like kinases	1 *Xoo* isolate	7	[47]
*OsCRK10*	Cysteine-rich-receptor-like kinases	1 *Xoo* isolate	7	[47]
*OsCDPK1*	Calcium-dependent protein kinases	*Xoo*	3	[48]
*OsILA1*	Raf-like MAPKKK	9 *Xoo* isolates	6	[49]
*lc7*	Ferredoxin-dependent glutamate synthase1	7 *Xoo* isolates	7	[50]
*OsLYP4*	Lysin motif-containing proteins	1 *Mo* isolate, 1 *Xoo* isolate, 1 *Xoc* isolate	9	[51]
*OsLYP6*	Lysin motif-containing proteins	1 *Mo* isolate, 1 *Xoo* isolate, 1 *Xoc* isolate	6	[51]
*OsWRKY67*	WRKY transcription factor	2 *Mo* isolates, 2 *Xoo* isolates	5	[52]
*IPA1*	Transcription factors	12 *Mo* isolates	8	[53,54]
*OsTFIIAα,*	Transcription factor IIA subunits	10 *Xoo* isolates, 6 *Xoc* isolates	5	[55]
*OsTFIIAβ*	Transcription factor IIA subunits	10 *Xoo* isolates, 6 *Xoc* isolates	5	[55]
*OsGLP2-1*	Germin-like protein	1 *Mo* isolate, 1 *Xoo* isolate	2	[56]
*OsSnRK1a*	Sucrose nonfermenting 1-related protein kinase 1	1 *Mo* isolate, 1 *Xoo* isolate, 1 *Cm* isolate and 1 *Rs* isolate	5	[57]
*OSK35* */OsSnRK1b*	Sucrose nonfermenting 1-related protein kinases	1 *Mo* isolate, 1 *Xoo* isolate	3	[58]
*OsCPK4*	Calcium-dependent protein kinase	1 *Mo* isolate, 1 *Xoo* isolate	2	[59]
*BSR1*	BIK1-like receptor-like cytoplasmic kinase	2 *Mo* isolates, 3 *Xoo* isolates, 1 *Bg* isolate, 1 *Cm* isolate, rice stripe virus	9	[60]
*OsBAG4*	Ubiquitin-like and BAG domain	1 *Mo* isolate, 1 *Xoo* isolate	1	[61]
*EBR1*	RING-Type E3 Ligase	1 *Mo* isolate, 6 *Xoo* isolates	5	[61]
*SPIN6*	Rho GTPase-activating protein (RhoGAP)	1 *Mo* isolate, 1 *Xoo* isolate	7	[62]
*OsWAK25*	Wall-associated kinases	2 *Mo* isolates, 1 *Xoo* isolate	3	[63]
*OsCUL3a*	Cullin 3-based RING E3 ubiquitin ligases	1 *Mo* isolate, 3 *Xoo* isolates	2	[64]
*OsDRP1E*	Dynamin-related protein	1 *Mo* isolate, 1 *Xoo* isolate	9	[65]
*SPL33*	Eukaryotic translation elongation factor 1 alpha (eEF1A)-like protein	12 *Mo* isolates, 11 *Xoo* isolates	1	[66]
*LMM5.1*	Eukaryotic translation elongation factor 1A (eEF1A)-like protein	6 *Mo* isolates, 5 *Xoo* isolates	1	[67]
*LMM5.4*	Eukaryotic translation elongation factor 1A (eEF1A)-like protein	6 *Mo* isolates, 5 *Xoo* isolates	4	[67]
*LML1*	Eukaryotic release factor 1 (eRF1) protein	4 *Mo* isolates, 6 *Xoo* isolates	4	[68]
*OsABA2*	Xanthoxin dehydrogenase	2 *Mo* isolates, 4 *Xoo* isolates	3	[69]
*SPL35*	CUE (coupling of ubiquitin conjugation to ER degradation) domain-containing protein	8 *Mo* isolates, 4 *Xoo* isolates	3	[70]
*OsHDT701*	Histone deacetylase	4 *Mo* isolates, 1 *Xoo* isolate	5	[71]
*OsMPK15*	Mitogen-activated protein kinase	2 *Mo* isolates, 2 *Xoo* isolates	11	[72]
*Bsr-k1*	Tetratricopeptide repeats (TPRs)containing protein	7 *Mo* isolates, 10 *Xoo* isolates	10	[73]
*OsALDH2B1*	Aldehyde dehydrogenase	1 *Mo* isolate, 1 *Xoo* isolate, 1 *Xoc* isolate	6	[74]
*OsPAL4*	Phenylalanine ammonia-lyase	1 *Mo* isolate, 1 *Xoo* isolate, 1 *Xoc* isolate	2	[75]
*OsHsfB4d*	Class B heat-shock factor	1 *Xoo* isolate, 1 *Xoc* isolate	3	[76]
*OsHsp18.0-CI*	Heat-shock proteins	5 *Xoc* isolates	3	[77]
*OsPAD4*	Phytoalexin-deficient 4	2 *Xoo* isolates, 1 *Xoc* isolate	11	[78]
*OsGRXS15*	Glutaredoxins family proteins	1 *Xoo* isolate, 1 *Ff* isolate	1	[79]
*OsNH5N16*	Pathogenesis-related genes 1 homologs (NHs)	1 *Xoo* isolates, 1 *Ff* isolate	11	[80]
*OsASR2*	Abscisic acid, stress, and ripening 2 protein	1 *Xoo* isolate, 1 *Xoc* isolate	11	[81]
*Os2H16*	Short-chain peptide-encoding protein	1 *Xoo* isolate, 1 *Xoc* isolate	6	[82]
*OsGF14e*	14-3-3 protein	1 *Xoo* isolate, 1 *Rs* isolate	2	[83]
*OsWRKY30*	WRKY transcription factors	1 *Mo* isolate, 1 *Rs* isolate	8	[84]
*OsACS2*	1-aminocyclopropane-1-carboxylic acid synthase	2 *Mo* isolates, 1 *Rs* isolate	4	[85]
*OsMESL*	Methyl esterase-like protein	1 *Mo* isolate, 1 *Xoo* isolate, 1 *Rs* isolate	7	[86]
*OsBON1*	Copine protein	1 *Mo* isolate, 3 *Xoo* isolates, 1 *Rs* isolate	2	[87]
*OsBON3*	Copine protein	1 *Mo* isolate, 3 *Xoo* isolates, 1 *Rs* isolate	5	[87]

^1^ The pathogens and number of isolates to which resistance is conferred by the genes in the first colum. *Burkholderia glumae*, *Bg*; *Cochliobolus miyabeanus*, *Cm*; *Magnaporthe oryzae*, *Mo*; *Rhizoctonia solani*, *Rs*; *Fusarium fujikuroi*, *Ff*; *Xanthomonas oryzae* pv. *oryzae*, *Xoo*; *Xanthomonas oryzae* pv. *oryzicola*, *Xoc*. ^2^ Chr: chromosome.
